# Polymorphisms in *ADRB2 *gene can modulate the response to bronchodilators and the severity of cystic fibrosis

**DOI:** 10.1186/1471-2466-12-50

**Published:** 2012-09-05

**Authors:** Fernando A L Marson, Carmen S Bertuzzo, Antônio F Ribeiro, José D Ribeiro

**Affiliations:** 1 Department of Pediatrics, School of Medical Sciences, University of Campinas, 13081-970, P.O. Box: 6111, Campinas, SP, Brazil; 2 Department of Genetics, Faculty of Medical Sciences, University of Campinas, 13081-970, P.O. Box: 6111, Campinas, SP, Brazil

**Keywords:** Beta 2 adrenergic receptor, *ADRB2* gene, Pulmonary function, Cystic fibrosis, Phenotype, Genotype

## Abstract

**Background:**

The most common cystic fibrosis (CF) manifestation is the progressive chronic obstructive pulmonary disease caused by deficiency, dysfunction, or absence of the CFTR (Cystic Fibrosis Transmembrane Regulator) protein on the apical surface of the cells in the respiratory tract. The use of bronchodilators (BD), and inhaled corticosteroids (IC) have been suggested for the management of airway inflammation in CF. The effectiveness of BD and IC have been verified, proven in laboratory and in the clinical treatment for asthma patients. However, in CF, the effectiveness of these drugs is controversial. The extent of asthma’s response to BD depends on the presence of polymorphisms in the *ADRB2* gene. In contrast, in CF, little is known about the response to the BD and the association of CF´s severity with the different polymorphisms in *ADRB2* gene. In this context, our objective was to verify whether the Arg16Gly and Glu27Gln polymorphisms in *ADRB2* gene are associated with severity and with the bronchodilator response in CF patients.

**Method:**

Cross-sectional study of 122 CF patients subjected to analysis of mutations in the *CFTR* gene, polymorphisms in *ADRB2* gene, along with clinical and laboratorial characteristics of severity.

**Result:**

The Arg16Gly polymorphism in *ADRB2* gene was associated with pancreatic insufficiency(p:0.009), Bhalla score(p:0.039), forced expiratory volume in the first second[FEV_1_(%)](p:0.003), forced expiratory flow between 25 and 75% of the forced vital capacity-FVC[FEF25-75(%)](p:0.008) and lower age at the first isolation of the *Pseudomonas aeruginosa*(p:0.012). The response to the BD spirometry was associated with clinical severity markers, FEV_1_(%)(p:0.011) and FEF25-75(%)(p:0.019), for the Arg16Gly polymorphism in the *ADRB2* gene. The haplotype analysis showed association with the FEV_1_/FVC marker from the spirometry test, before and after using the BD, with higher values in the group with Gly/Gly and Glu/Glu, respectively, for the Arg16Gly and Gln27Glu polymorphisms. The analysis by MDR2.0 software, showed association with FEF25-75%; the response to Arg16Gly was respondent by 17.35% and Gln27Glu by 6.8% in variation found.

**Conclusion:**

There was an association between the Arg16Gly and Gln27Glu polymorphisms in *ADRB2* gene with CF´s severity and bronchodilator response.

## Background

Studies have confirmed the mechanisms though which cystic fibrosis (CF) patients with similar mutations show significantly different signs and symptoms [1-4]. Even among homozygous for classes I, II and/or III mutations, the disease's clinical expression may be very different. Explanations for these broad clinical variations included environmental factors [5], medical management [3], nutrient intake [6], psychological [7], economic situations [3], and classes of mutations and polymorphisms in modifier genes [2-4].

Unlike most patients with asthma, the inflammation in CF is predominantly neutrophilic [8], with an intense inflammatory component and lower rate of bronchial hyper responsiveness [9]. In contrast, inhaled bronchodilator (BD) has been used for the management of these two conditions [10]. In addition to the BD, β-agonists affect the other functions of the airways: they reduce inflammatory cells mediators release, cholinergic neurotransmission and vascular permeability, as well as increase the clearance mucociliary [11], features that justify its use in CF.

Studies regarding the BD asthma response have revealed different results, depending on the polymorphism in *ADRB2* gene [12]. The physical and chemical characteristics of the β2 (β2-AR) receptors are found in scientific literature, and demonstrate its importance to many biological processes, including muscle relaxation in the lungs [13-15]. Among polymorphisms identified in coding region of the *ADRB2* gene, the Arg16Gly (c.46A > G) and Gln27Glu (c.79C > G) are the most studied in asthma [12]. The Gln27Glu polymorphism confers resistance from the ADRB2 protein to BD action. The *ADBR*2*G allele is protective against asthma, reducing its risks by 27% [13]. ADBR2*G induces changes in receptor regulation, which occurs due to its increased susceptibility to the protein's degradation.

Haplotypes that include the ADBR2*A allele have better response to the inhaled β2 agonists. Presumably, better response to exogenous agonists reflects better response to endogenous agonists and a protective effect against asthma and other bronchial hyper responsiveness diseases [16]. Haplotypes with two Arg16Gly/Gln27Glu mutant receivers showed that the effects of the Arg16Gly predominate over the Gln27Glu. These receptors are subject to greater regulation by agonists than the normal allele from the β2-AR receptor [13].

The effectiveness of BD and IC have been attained and proven in both the laboratory and the clinical treatment for asthma patients. Nevertheless, with CF the effectiveness of these drugs is controversial. The effectiveness of asthma’s response to the BD is dependent on polymorphisms in *ADRB2* gene*.* In contrast, with CF, little is known about the response to the BD and the CF´s severity in association with the different polymorphisms in *ADRB2* gene [17-19]. Although previous studies had showed an association between bronchodilator response and the polymorphisms in the *ADRB2* gene, these studies did not take into account secondary clinical variables that can provide extra information about the mechanism associated with action of the ADRB2 protein.

Recently, it was discovered that the regulation of CFTR activity is mediated by the activation of G-coupled receptors such as the β_2_-AR. CFTR and the β_2_-AR protein are associated in a large macromolecular complex [20-22]. Taouil and colleagues showed that the long-acting salmeterol treatment increased wild-type CFTR expression in primary human airway epithelial cells. They demonstrated that wild-type *CFTR* levels increased more than 2-fold during this treatment, while the mRNA levels were unaffected [21].

In this context, the aim of this study was to investigate the influence of the Arg16Gly and Glu27Gln polymorphisms from the *ADRB2* gene on 26 clinical severity markers and the bronchodilator response in CF patients from a university referral center.

## Methods

A cross-sectional study was conducted in a referral center for CF treatment, at the Campinas University Hospital (UNICAMP) in 2010 and 2011. A total of 122 patients were included, with at least seven years of age and who underwent pulmonary function tests before and after the use of inhaled Albuterol (400 mg) according to the American Thoracic Society (2011) [23]. The patients underwent two perspiration tests of chlorine and sodium with chlorine levels equal to or greater than 60 mEq/L, and/or identification of two mutations in *CFTR* gene [F508del, G542X, G551D, R553X, R1162X, I618T and N1303K]. The project was approved by the University Ethics Committee (#528/2008), all patients and/or their guardians signed an informed consent.

We clinically evaluated the patients according to the following clinical severity markers: clinical scores (Shwachman-Kulczycki, Kanga and Bhalla) [24]; body mass index (BMI) [for the patients older than 19 years the BMI = weight/(height)^2^ formula was used; for the remaining patients the WHO ANTHRO PLUS (children 7-19 years old) [25]; patient age (group: younger ≤ 24 months and older > than 24 months) and age at diagnosis (according to the sodium and chloride in altered perspiration - younger age group ≤ 24 months and older > than 24 months); first clinical symptoms [(digestive - younger age ≤ 3 months and older > than 3 months; pulmonary - younger age ≤ 6 months and older > than 6 months)]; the period up to 1st colonization by *Pseudomonas aeruginosa* (younger ≤ 31 months and older > than 31 months); presence of microorganisms in the sputum *(P. aeruginosa* mucoid and non *mucoid, Achromobacter xylosoxidans, Burkolderea cepacia* and *Staphylococcus aureus*)*;* transcutaneous oxygen saturation; pulmonary function tests before and after BD use and CF´s co morbidities (nasal polyps, osteoporosis, meconium ileus, diabetes mellitus and pancreatic insufficiency).

The Spirometry proof was performed using a speedometer model CPFS/D (Med Graphics, Saint Paul, Minnesota, USA). Data were recorded by the BREEZE PF Version 3.8 B software for Windows 95/98/NT with inclusion of the following markers: forced vital capacity - FVC(%), forced expiratory volume in the first second - FEV_1_(%), ratio between the FEV_1_ and the FVC, and forced expiratory flow between 25-75% of the FVC (FEF25-75%). All patients underwent spirometry test before and after 15 minutes of the BD - albuterol (400 mg) administration. Patients receiving the BD therapy were instructed to stop the medication 8 hours prior to the short-acting beta agonist spirometry test and 48 hours prior to the long-acting beta agonist [23].

Genomic DNA was extracted using phenol-chloroform from venous blood samples. Polymorphism analysis in modifier genes was performed by the polymerase (PCR) allele specific (ARMS) reaction, according to Littlejohn et al. (2002) [26]. Four reactions were performed, (ARMS1a, ARMS2a, and ARMS1b ARMS2b), each containing a common primer and one specific allele. All four reactions were performed under the same conditions. Each consisting of a total of 10μL containing 1x4 PCR buffer, 200 μM dNTPs, 5.0nM MgCl2, 0.4U of Taq polymerase, 0.2 pMoles of each primer and 1.0μL (approximately 50 ng) of genomic DNA. The PCR amplification conditions consisted of 5 min at 94°C followed by 35 cycles of 94°C for 1 min, 60°C (46 A or G - 16 Arg or Gly), or 67°C (70°C or G - 27 Gln or Glu), 72°C for 1 minute and 72°C for 10 min. The amplicons were submitted to electrophoresis on 12% acryl amide gel and stained with ethidium bromide for visualization of the results.

### Statistical analysis of the data

Statistical analysis was performed by the software SPSS v.17.0 (SPSS Inc., Chicago, IL, USA) [27], Epi Info v.6.0 [28] and R version 2.12 (Comprehensive R Archive Network, 2011). The statistical power calculation for the sample was performed by the GPOWER 3.0.5 software [29] demonstrating statistical power above 80% for the conducted analysis. Alpha = 0.05 was used in all data analyses.

Analysis of variance (ANOVA) and chi-squared (^2^) - Odds Ratio (OR) tests were performed for quantitative and categorical variables, respectively. Pulmonary function comparison data before and after the BD use was performed by the ANOVA test; subsequently, the different genotypes for the Arg16Gly and Gln27Glu polymorphisms in *ADRB2* gene were analyzed by the Student’s *t* test. Supplementary analysis performed by MDR software 2.0 (Multifactor dimensionality Reduction - Norris-Cotton Cancer Center, 2008) and MDRPT 0.4.7 (Permutation Test Multifactor dimensionality Reduction - Norris-Cotton Cancer Center, 2008), to evaluate the influence of both polymorphisms with the variation in the spirometry values of the bronchospasm response. The analyses of subgroups by age were not possible in our population as subgroups had a small number of patients.

In order to avoid spurious data due to the multiple tests problematic [30], the significance level α was adjusted by the Bonferroni correction (α corrected = 0.05/number of tests).

## Results

The frequencies of each genotype for the Arg16Gly polymorphism was: 23(18.85%), 54(44.26%) and 45(36.89%), respectively for the Arg/Arg, Arg/Gly and Gly/Gly. For the Gln27Glu polymorphism was 61(50.00%), 51(41.80%) and 10(8.20%), respectively for the Gln/Gln, Gln/Glu and Glu/Glu. The population is within the Hardy-Weinberg equilibrium (^2^ = 0.88, p > 0.05; ^2^ = 0.02; p > 0.05, respectively for the Arg16Gly and Gln27Glu polymorphisms in *ADRB2* gene). The patients' characteristics included in the study are described in Table 1.

**Table 1:** **Characteristics of patients included in the study (N = 122)**^**1**^

**Male**	**48.8 %**
Age	246.68 ± 168,73 months (87 - 932 months)
Caucasoid	93.4%
BMI - Thinness and Thinness accentuated	22.3%
SaO2	94.87 ± 4.53 (66 – 99)
Bhalla	9.41 ± 5.57 (0 – 25)
Kanga	19.37 ± 5.01 (11 – 40)
Shwachman-Kulczycki	65.41 ± 16.02 (20 – 95)
FVC (%)	78.27 ± 22.86 (19 – 135)
FEV_1_ (%)	70.28 ± 26.17 (17 – 125)
FEV_1_/FVC (%)	83.83 ± 15.79 (37 – 137)
FEF25-75%	58.50 ± 34.83 (7 – 150)
FVC (%) reversibility	0.92 ± 10.48 (-27 – 32)
FEV_1_ (%) reversibility	2.15 ± 9.45 (-12 – 31)
FEV_1_/FVC (%) reversibility	2.84 ± 9.69 (-19 – 47)
FEF25-75% reversibility	7.24 ± 9.43(-12 – 30)
Nasal Polyps	21.7%
Diabetes mellitus	20.8%
Osteoporosis	20.8%
Pancreatic insufficiency	76%
Meconium ileus	9.1%
*P. aeruginosa* status ^2^	53.7%
*P. aeruginosa* mucoid status ^2^	45.5%
*B. cepacia* status ^2^	9.1%
*A. xylosoxidans* status ^2^	9.9%
*S. aureus* status ^2^	78.5%
*CFTR* mutation	
F508del/F508del	29 (24%)
F508del/G542X	10 (8.3%)
F508del/N1303K	3 (2.5%)
F508del/R1162X	3 (2.5%)
F508del/R553X	1 (0.8%)
G542X/I618T	1 (0.8%)
G542X/R1162X	1 (0.8%)
F508del/No identified mutation	26 (21.5%)
G542X/No identified mutation	4 (3.3%)
No identified mutation	43 (35.3%)

*N *- Sample size; *BMI *- body mass index; % - percentage; *FVC* - forced vital capacity; *FEV*_*1*_ - forced expiratory volume in the first second; FEF25-75% - forced expiratory flow between 25 and 75% of CVF. 1. Continuous variables expressed as mean ± SD (range). 2. Based on 3 consecutive positive respiratory cultures.

**Figure 1:**
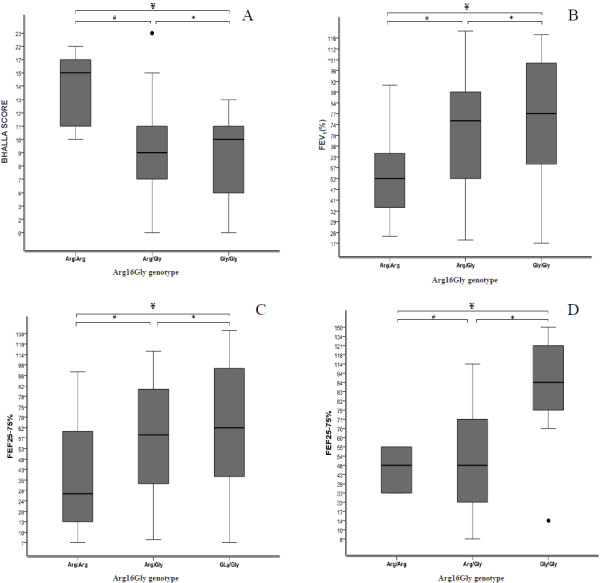
**Bhalla score and spirometry values in Cystic Fibrosis patients modulated by polymorphism in *ADRB2 *gene. **Association of the cystic fibrosis severity with polymorphism Arg16Gly in *ADRB2 *gene. **A**. Bloxplot of the Bhalla score in patients with two identified mutations in *CFTR *gene. The Bhalla score data is a gross value. # p:0.011; * p:0.779; ¥ p:0.007. **B**. Bloxplot of FEV_1_(%) in patients without taking CFTR mutation into account. # p:0.007; * p:0.277; ¥ p:0.003. **C**. Bloxplot of FEF25-75% in patients without without taking CFTR mutation into account. # p:0.038; * p:0.43; ¥ p:0.015. **D**. Bloxplot of FEF25-75% in patients with no mutations identified in *CFTR *gene. # p:0.933; * p:0.008; ¥ p:0.001. The data of the spirometry are in percentage of the predicted population value. The test used for the analyses was the T´student test.

The Arg16Gly polymorphism in *ADRB2* gene, without the distribution by the *CFTR* gene genotype, was associated with the FEV_1_(%) (p = 0.003) and FEF25-75% (p = 0.008), the minor value was observed in patients with Arg/Arg genotype. Patients with no identified mutation in *CFTR* gene were associated with better value to FEF25-75% to Gly/Gly genotype (p = 0.008). The group with two mutations identified in *CFTR* gene was associated with bigger value to Bhalla score to Arg/Arg genotype (p = 0.04) (Figure 1). In this same patients’ group there was association with the pancreatic insufficiency, and the Arg/Arg genotype with an OR of 0.13 (95%CI = 0.03-0.86) and Gly/Gly with an OR of 8 (95%CI = 1.95-67.7) with the presence of co morbidity and with the first isolated *P. aeruginosa,* OR of 0.22 (95%CI = 0.05-0.99) for the Gly/Gly genotype.

The Gln27Glu polymorphism in *ADRB2* gene showed no statistically significant association with the variables used as CF´s severity markers in this study.

**Figure 2:**
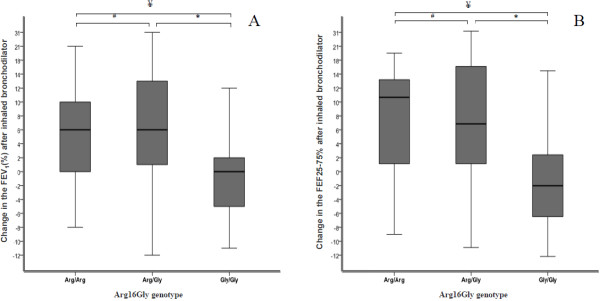
**Bronchodilatador response in Cystic Fibrosis patients modulated by polymorphism in *ADRB2 *gene. **Response to the inhaler bronchodilators by the alteration of spirometry markers values in the Cystic Fibrosis patients according to polymorphism Arg16Gly in *ADRB2* gene. **A**. Bloxplot of the variation in the FEV_1_(%) before and after using bronchodilators in patients without without taking CFTR mutation into account. # p:0.361; * p:0.001; ¥ p:0.002. **B**. Bloxplot with the variation in the FEF25-75(%) before and after using bronchodilators in patients without without taking CFTR mutation into account. # p:0.031; * p:0.026; ¥ p:0.01. Analysis made group by group by the T´student test. The data from the pulmonary function test are in percentage of the predicted population value.

The analysis of the polymorphisms in *ADRB2* gene and their association with BD mediated bronchospasm response occurred for the Arg16Gly polymorphism (Table 2). The variables demonstrating modulations were: FEV_1_(%) (p = 0.01) and FEF25-75% (p = 0.02) (Figure 2). The Gly/Gly genotype was associated with a minor bronchodilator response. The variation values data for the spirometry test markers according to the haplotypes in the *ADRB2* gene for the Arg16Gly and Gln27Glu polymorphisms are described in Table 3. In the haplotypes analysis the groups with the GG genotype were considered in at least one, both, or in none of the polymorphisms analyzed.

**Table 2:** **Association of polymorphisms in *ADRB2 *gene with spirometry of Cystic Fibrosis patients**^**1**^

**Variable**	**Arg16Gly**					**Gln27Glu**				
	**Arg/Arg**	**Arg/Gly**	**Gly/Gly**	**F**	**p**	**Gln/Gln**	**Gln/Glu**	**Glu/Glu**	**F**	**p**
FVC (%)	23 (82.04 ± 4.48)	54(79.30 ± 3.21)	44(75.05 ± 3.43)	3.25	0.08	63(77.9 ± 2.92)	48(79 ± 3.37)	10(77.1 ± 6.48)	0.02	0.90
FEV_1_ (%)	**23(72 ± 5.57)**	**53(72.57 ± 3.47)**	**44(66.64 ± 4.10)**	**6.80**	**0.01**	62(70.34 ± 3.49)	48(70.75 ± 3.63)	10(67.70 ± 8)	0.45	0.50
FEV_1_/FVC	23(82 ± 4.27)	52(84.79 ± 1.82)	44(83.33 ± 2.44)	1.12	0.29	62(85.32 ± 1.9)	47(82.21 ± 2.48)	10(82.20 ± 4.96)	0.17	0.68
FEF25-75%	**23(58.17 ± 7.22)**	**53(59.91 ± 4.48)**	**44(56.98 ± 5.75)**	**5.82**	**0.02**	62(61.34 ± 4.64)	48(55.88 ± 4.84)	10(53.50 ± 9.99)	1.23	0.27

% - Percentage; *FVC* - forced vital capacity; *FEV *- forced expiratory volume; *FEF*25-75% - forced expiratory flow between 27-75% of the FVC. 1. Continuous variables expressed as number of patients (mean ± SD (range). Statistical analysis conducted by analysis of variance (ANOVA) through (in bold) statistically significant values with less than 0.05.

**Table 3:** **Change in FVC, FEV**_**1, **_**and FEF25-75 FEF1/FVC post-bronchodilator by *ADRB2 *haplotype**^**1**^

	**Arg16Gly/Gln27Glu GG haplotype group**			**p- value**
	**0 GG (n = 56)**	**1 GG (n = 34)**	**2 GG (n = 4)**	
Change in FVC(%)	-0.17 ± 9.86	2.29 ± 11.87	5.25 ± 4.03	0.17
Change in FEV_1_(%)	2.10 ± 9.42	2.18 ± 9.57	2.75 ± 11.41	0.92
Change in FEV_1_/FVC(%)	**4.61 ± 10.45**	**0.53 ± 7.75**	**-2.25 ± 9.61**	**0.02**
Change FEF25-75(%)	6.36 ± 30.75	8.62 ± 41.75	8 ± 39.45	0.79

% - Percentage; *FVC *- forced vital capacity; *FEV* - forced expiratory volume; *FEF*25-75% - forced expiratory flow between 27-75% of the FVC. Statistical analysis performed by linear regression. 1. Percent change calculated as 100 x (post-bronchodilator value – pre-bronchodilator value)/pre-bronchodilator value.

The statistical analysis performed by the MDR 2.0 program in comparison with the curve stipulated by the data held by the MDRPT 0.4.7 program, demonstrated the influence of the two polymorphisms analyzed with the variance of FEF25-75% before and after using the BD [Bal Acc. testing.: 0.772, p: 0.0034, ratio: 0.9934]. From the observed variation for the FEF25-75%, according to the test performed, the Arg16Gly polymorphism is responsible for 17.4% and the Gln27Glu for 6.8% of the variation.

## Discussion

In the last decade, several studies have shown that the receptor for beta 2 agonist is intimately connected, physically and functionally, to the CFTR protein on the surface of the epithelial cells in the airways [20]. Interestingly, the function of this complex behaves differently if the stimulus is caused by long or short term beta 2 agonist substances [20-22]. The clinical response of the CF patients in relation to the long-term beta agonists in the different polymorphisms in *ADRB2* gene has not been studied, while for the short term use, there are few studies in the literature [17-19]. With asthma, the Arg16Gly and Gln27Glu polymorphisms in *ADRB2* gene cause different reactions to the Bronchodilation promoted by BD [12]; however, little is known about their impact on the severity and response to Bronchodilation caused by CF BD [17-19].

To the best of our knowledge it is known that the allele frequency for the Arg16Gly and Gln27Glu polymorphisms in *ADRB2* gene is different in adults and children with CF [19]. Adult patients with CF have a higher frequency for the Gly16 and Glu27 alleles compared to people CF free. Children with CF have a lower frequency for Gly16 and similar with the Glu27. Young adults with CF in relation to the variant forms in *ADRB2* gene did not differ from the population in general. In our population there was no difference in genotype distribution for the Arg16Gly (p:0.92) and Gln27Glu (p:0.88) polymorphisms in *ADRB2* gene relative to the patients' age group.

Our study showed an association of the Arg16Gly polymorphism in *ADRB2* gene with the severity of CF markers. The variation in the spirometry proof before and after the use of BD was associated with the Arg16Gly and Gln27Glu polymorphisms in *ADRB2* gene. Twenty six of CF´s severity markers were used in the study; and the response to Bronchodilation was assessed by four spirometry markers.

Hart et al. (2005) [18] used the FEV_1_(%) and the FEF25-75% as severity markers, in addition to the following variables adjustment: age, gender, the F508del homozygous and *Pseudomonas aeruginosa* status. Buscher et al. (2002) [17] used the following markers: FEV_1_, FVC, flow at lower lung volumes (MEF50%VC), measurement of cAMP accumulation in intact mononuclear lymphocytes, age, gender, BMI, F508del homozygous and *Pseudomonas aeruginosa* status. Hart et al. (2005) [18] considered the presence of values 12% higher in the FEV_1_ after using the BD, (the examination of spirometry occurred 15 minutes after administration of BD), a positive response to Bronchodilation, while Buscher et al. (2002) [17] believed in the 10% variation for the same marker (the Spirometry test occurred 5 minutes after BD administration). Steagall et al. (2007) [19] used as markers: age, sex, F508del status, carbon monoxide diffusing capacity (DLCO), FEV_1_(%) and the FVC(%). The positive response to Bronchodilation was seen as a positive variation superior to 12% for the FEV_1_ and the FVC spirometry markers.

The presence of different genotypes in the analyzed mutations, (belonging to the classes I, II and/or III), of the *CFTR* gene, along with the polymorphisms in *ADRB2* gene were associated to different markers related to CF´s severity.

An intriguing aspect of our study, not previously described, were the findings that CF´s patients with two mutations from classes I, II and/or III, Arg/Arg homozygous for the Arg16Gly polymorphism, have greater impairment of lung function than the Gly/Gly homozygous, provided by the Bhalla score. And the Gly/Gly homozygous showed higher pancreatic insufficiency than the Arg/Arg. The two clinical variables are associated with CF severity, and are modulated by the Gly/Gly genotype to Arg16Gly polymorphism in our population. The Gly allele is associated with fast protein degradation [13] that was identified as a severity factor.

The first isolation of the *P. aeruginosa* was confirmed later in patients with the Gly/Gly genotype and two mutations classes I, II and/or III in *CFTR* gene. The early colonization is associated with clinical deterioration and a greater degree of airway inflammation [31].

The response to this polymorphism and the clinical aspects of CF´s patients has not been studied in the literature referenced, except for Buscher et al., (2002) [17], whose analysis of some of the markers, (age, gender, BMI and *P. aeruginosa* status), showed no significant association, a fact that hampered data comparison. Another relevant fact is the Gln27Glu polymorphism lack of association with severity markers in the study. There were no references in the scientific literature indicating this polymorphism as a modulator of CF´s severity.

Our results show that CF´s patients with the Arg/Arg genotype from the Arg16Gly polymorphism have lower values of the FEV_1_ and FEF25-75%. The observation contrasts with the data from Buscher et al. (2002) [17] who reported higher values of the Arg/Arg genotype in 87 CF´s patients for the FEV_1_, FVC, and MEF50%VC markers, when compared with subjects with a genotype containing at least one Gly allele. The same was observed when the genotype for the delF508 mutation was used; 51 patients were analyzed, and higher values for the markers were found for the same genotype.

In our data the Gln27Glu polymorphism in *ADRB2* gene was not associated with spirometry markers. Diversely, in the Buscher et al. (2002) [17] study, the Gln/Gln genotype for the Gln27Glu polymorphism, when compared with the others, showed a higher correlation for the FEV_1_, FVC, and MEF50%VC values. In the Steagall et al. (2007) [19] study there was no correlation between the DLCO, FEV_1_ and the FVC in patients with homozygous F508del stratified by the Arg16Gly and Gln27Glu polymorphisms genotype in *ADRB2* gene.

The patients with no identified mutations in *CFTR* gene, in our study, showed higher values in the FEF25-75% for the Gly/Gly genotype. Thus, comparing the data discussed above, we denote the influence of the Arg16Gly polymorphism in *ADRB2* gene that occurs in the spirometry, and that is also influenced by the genotype in the *CFTR* gene.

We found that the Arg27Gly polymorphism in *ADRB2* gene was associated with the bronchospasm response mediated by beta 2 agonists. The FEV_1_(%) variation was lower in patients with the Gly/Gly genotype, when compared with the Arg/Arg and Arg/Gly genotypes. The FEF25-75% was lower in patients with the Gly/Gly genotype when compared to the Arg/Arg and Arg/Gly. Patients with the Arg/Arg genotype showed higher FEF25-75% when compared to the Arg/Gly genotype.

In the study by Hart et al. (2005) [18], one hundred and six patients with CF underwent spirometry before and after the BD administration, and the Arg16Gly and Gln27Glu polymorphisms genotype was determined. The positive response was observed in 15% (23/154) of the included patients and 17% (18/106) among the patients who had genotype for the gene *ADBR2* determined. There was no significant relationship between the *ADRB2* gene genotype for the Arg16Gly and Gln27Glu polymorphisms and the response to the BD [18]. However, after the FEV_1_ adjustment in percentage for the predicted and the status of infection by the *P. aeruginosa,* a significant difference occurred to the FEF25-75% percentage of change (4.9%, 18.0% and 22.4% for the genotypes AA, AG and GG; respectively). The FEF25-75% percentage of change showed no substantial difference for the codon 27, even after the adjustment for the infection by *P. aeruginosa* and by the predicted baseline of FEV_1_(%) [18]. After adjusting the FEV_1_ percentage predicted baseline, the codon 27 groups differed significantly from the percentage of change in the FEV_1_(%) (4.1%, 8.1% and 9.3% for the genotypes CC, CG and GG; respectively) [18]. The authors suggested that the effect of the two polymorphisms were complex and the haplotype analysis may be more informative than any genotype alone [18]. To that effect, a large number of patients would be needed, only possible in multi-centric studies. Then again, in multi-centric studies the environment and diverse management systems could provide significant bias in the clinical severity outcome.

In the study by Buscher et al. (2002) [17], no statistically significant difference was found for the FEV_1_ average value and in the absolute number of patients with positive response to BD among the Arg16Gly polymorphism genotypes in *ADRB2* gene.

In the Steagall et al. (2007) [19] study homozygous patients with the delF508 mutation were analyzed for their reactions to Bronchodilation. Patients who showed no response to the Bronchodilation had, on average, higher values in the FVC and FEV_1_ for the Arg/Arg genotype in the Arg16Gly polymorphism of the *ADRB2* gene when compared to other genotypes; however, in the analyzed group there were only two patients with the Arg/Arg and two with the delF508 mutations for the *CFTR* gene. For the Gln27Glu polymorphism, the Gln/Gln genotype was associated with higher FVC values. In the responders group only the Gln27Glu polymorphism was associated with higher values of the DLCO, FVC and FEV_1_ for the Gln/Glu genotype. In our study, the distribution of responders and non-responders to the pulmonary function test with the BD use, and the distribution by the polymorphisms in *ADRB2* gene and mutations in classes I, II and/or III, was performed, but the sample power was less than 80%.

The long term decline in spirometry values was not evaluated in this study, even though it presents a promising field of study, verified by Buscher et al. (2002) [17] who demonstrated that, in 59 CF patients aged 6-20 years, the biggest FEV_1_ decline was in patients with at least one Gly allele, while no change in pulmonary function was seen in patients homozygous for Arg.

The genic interaction analysis performed by the MDR 2.0 program showed a combination of both polymorphisms with the variation for the FEF25-75% but not to other markers of severity. Thus, this corroborates that the polymorphisms in *ADRB2* gene influence the Bronchodilation response.

In the haplotypes analysis the Ar/Arg + Gln/Gln presence was associated with the best response to Bronchodilation for the marker FEV_1_/FVC (N = 56; +4.61% ± 10.45), when compared to the other groups: 1 GG [Arg/Arg + Glu/Glu, Arg/Gly + Glu/Glu, Gly/Gly + Gln/Gln, Gly/Gly + Gln/Glu (N = 34; +0.53% ± 7.75)] and 2 GG [Gly/Gly + Glu/Glu (N = 4; -2.25% ± 9.605)]. In the study by Hart et al. (2005) [18], the haplotypes analysis for three different models demonstrated association with the FEV_1_ marker. And the values of 4.4 (± 5.8), 7.9 (± 8.7) and 9.0 (± 5.8); respectively to 0 GG (N = 27), 1GG (N = 64) and 2GG (N = 15), with *p* values equal to 0.033, 0.027 and 0.305; respectively for the models, additive, dominant and recessive.

In our results, the bronchodilator response was reduced with the presence of the variants with Gly or Glu allele, a fact contrary to that reported by Hart et al. (2005) [18].

Possible limitations of our study included: sample size, different age groups, and different severities of lung disease that could express differences in the BD response.

Our data can provide better understanding about the association between polymorphism associated with β_2_-AR protein and the clinical markers of CF´s severity, and others studies about the large macromolecular complex are necessary to improve knowledge about the interaction of the proteins on the bronchial epithelial surface.

## Conclusion

The influence of polymorphisms in the CF´s severity and bronchospasm response is controversial in the scientific literature, and seldom studied. Our results support the idea that differences in the degree of airway responsiveness to the BD may be associated with the *ADRB2* gene genotype in the CF. Arg16Gly and Gln27Glu polymorphisms are associated with the response to the Bronchodilation. For the spirometry and other clinical variables of severity; the Arg16Gly polymorphism showed statistically significant association; diversely, the Gln27Glu showed no association. The Arg/Arg genotype to Arg16Gly polymorphism was associated with a better spirometry to FEV_1_(%) and FEF25-75% clinical markers and minor risk to PI, in comparison with other genotypes. To bronchodilator response the haplotype data to the polymorphism show association with positive response to beta 2 agonists to absence of genotypes Gly16Gly and Glu27Glu to FEV_1_/FVC(%) marker.

We must pay attention to the need for more studies; with the suppressed polymorphisms and their association with the clinical severity variables using a larger number of CF´s patients with the same *CFTR* genotype for the mutation classes I, II and/or III; also patients with the same degree of change in the spirometry test to analyze the response to Bronchodilation.

## Competing interests

The authors declare that they have no competing interests.

## Authors’ contributions

FALM: made substantial contributions to conception and design, or acquisition of data, or analysis and interpretation of data; involved in drafting the manuscript or revising it critically for important intellectual content. CSB: carried out the molecular genetic studies and drafted the manuscript. AFR: has been involved in drafting the manuscript or revising it critically for important intellectual content. JDR: has given final approval of the version to be published. All authors read and approved the final manuscript.

## Pre-publication history

The pre-publication history for this paper can be accessed here:

http://www.biomedcentral.com/1471-2466/12/50/prepub
